# Temporal clinical trajectories and Day+14 landmark stratification of infection risk after CD19 CAR T-cell therapy

**DOI:** 10.3389/fcimb.2026.1933555

**Published:** 2026-10-23

**Authors:** Liuyang Yang, Liyu Shan, Shuai Feng, Renbin Zhao, Yan Wen, Xuezhong Gu, Zengzheng Li, Xiangmei Yao, Yajie Wang

**Affiliations:** 1 Department of Hematology, The First People’s Hospital of Yunnan Province, Affiliated Hospital of Kunming University of Science and Technology, Yunnan Province Clinical Research Center for Hematologic Disease, Kunming, China; 2 Yunnan Provincial Clinical Medical Center for Blood Diseases and Thrombosis Prevention and Treatment, The First People’s Hospital of Yunnan Province, Kunming, China; 3 Yunnan Atherosclerosis Cooperation Base of Chinese and Western Medicine, The First People’s Hospital of Yunnan Province, Kunming, China; 4 The Third Affiliated Hospital of Kunming Medical University, Yunnan Cancer Hospital, Peking University Cancer Hospital Yunnan, Yunnan, China; 5 The Second Affiliated Hospital of Kunming Medical University, Yunnan, China

**Keywords:** CAR-T, infection, landmark analysis, registry classification, transportability

## Abstract

**Background:**

Infection after CD19-directed chimeric antigen receptor T-cell (CAR-T) therapy develops over a changing clinical course. We examined two related questions: how completed Day+100 registry data characterize the infection-associated clinical phenotype, and whether information available by Day+14 can stratify subsequent infection risk through Day+100.

**Methods:**

We analyzed 3,302 adults with large B-cell lymphoma using a 70:30 development/test partition. An all-history classifier used completed Day+100 records. The corrected Day+14 cohort included 2,876 patients with observation extending beyond the landmark; models used information available by Day+14. Supplemental outer cross-validation repeated development, and logistic recalibration used development cross-fitted scores. A separate common-feature model was evaluated in 465 pediatric and young-adult patients with B-cell acute lymphoblastic leukemia.

**Results:**

Infection was recorded in 735/3,302 adults (22.3%). The all-history test AUROC was 0.724 (95% CI, 0.685–0.765). In the corrected Day+14 test set (862 patients; 113 infections), the dynamic-model AUROC was 0.669 (95% CI, 0.616–0.716) and AUPRC was 0.217 (95% CI, 0.165–0.286). At score cutoff 0.25, the low-score group contained 160/862 patients (18.6%) and 5 infections (3.1%; 95% CI, 1.0–7.1%). Its raw and recalibrated Brier scores were 0.198 and 0.110, compared with 0.114 for the development-prevalence reference. In the cross-disease evaluation, the common-feature model achieved an AUROC of 0.663.

**Conclusions:**

Completed records characterize infection-associated clinical trajectories. Day+14 information identifies a low-score subgroup for prospective evaluation of outpatient monitoring, with its coverage, event rate and calibration defining the conditions for that evaluation.

## Introduction

CD19-directed chimeric antigen receptor T-cell (CAR-T) therapy offers durable responses in relapsed or refractory large B-cell lymphoma (Neelapu et al., 2017; Schuster et al., 2019). Infection is concentrated in the first month after infusion, when lymphodepletion, cytopenias and immune-effector-cell toxicities overlap (Hill et al., 2018; Vora et al., 2020; Wudhikarn et al., 2020; Mikkilineni et al., 2021; Wudhikarn et al., 2025). Understanding how this changing clinical course relates to infection can inform the design of outpatient follow-up studies.

Baseline health, prior infection and treatment burden capture part of the variation in infection outcomes. Cytokine release syndrome (CRS), immune effector cell-associated neurotoxicity syndrome (ICANS), corticosteroid exposure and delayed immune recovery add information during follow-up (Hill et al., 2018; Park et al., 2018; Hill and Seo, 2020; Vora et al., 2020; Wudhikarn et al., 2020; Baird et al., 2021; Mikkilineni et al., 2021; Rejeski et al., 2022; Wat and Barmettler, 2022; Wudhikarn and Perales, 2022; Sutherland et al., 2025; Wudhikarn et al., 2025). Their interpretation depends on when each measurement becomes available relative to infection.

We examined two questions. The all-history analysis used completed Day+100 records to characterize combinations of clinical features associated with infection during that interval. The Day+14 landmark analysis evaluated whether information available at Day+14 predicted subsequently recorded infection through Day+100 among patients still under observation (Dafni, 2011; Collins et al., 2015; Wolff et al., 2019; Collins et al., 2024). The former describes a clinical phenotype; the latter defines a prognostic analysis with a fixed prediction time. We also evaluated transfer of a common-feature classifier from adult lymphoma to pediatric and young-adult leukemia. The Day+14 model defines a low-score subgroup for prospective evaluation of differentiated outpatient monitoring after CAR-T therapy.

## Methods

### Study design and source cohorts

This retrospective secondary analysis used two deidentified public-use datasets from the Center for International Blood and Marrow Transplant Research (CIBMTR), associated with published CAR-T infection studies (Rangarajan et al., 2025; Wudhikarn et al., 2025). The adult large B-cell lymphoma dataset supplied 3,332 numeric patient records; 30 lacked a usable binary infection endpoint, leaving 3,302 for analysis. The second dataset supplied 465 evaluable children, adolescents and young adults with B-cell acute lymphoblastic leukemia treated with tisagenlecleucel. The source studies covered December 2017 to June 2022 for adult lymphoma and 1 September 2017 to 30 June 2022 for leukemia (Rangarajan et al., 2025; Wudhikarn et al., 2025). The current public analysis subsets contained 118 and 71 distinct center identifiers, respectively; their individual infusion calendar dates were not released.

The all-history analysis classified infection through Day+100 from completed clinical records. The Day+14 analysis used information available at the landmark to predict infection during Days +15 to +100 among adults who were infection-free and still under observation at Day+14. Cross-disease evaluation applied a common-feature model developed in the adult lymphoma cohort to the pediatric and young-adult leukemia cohort (**Figure 1**).

**Figure 1:**
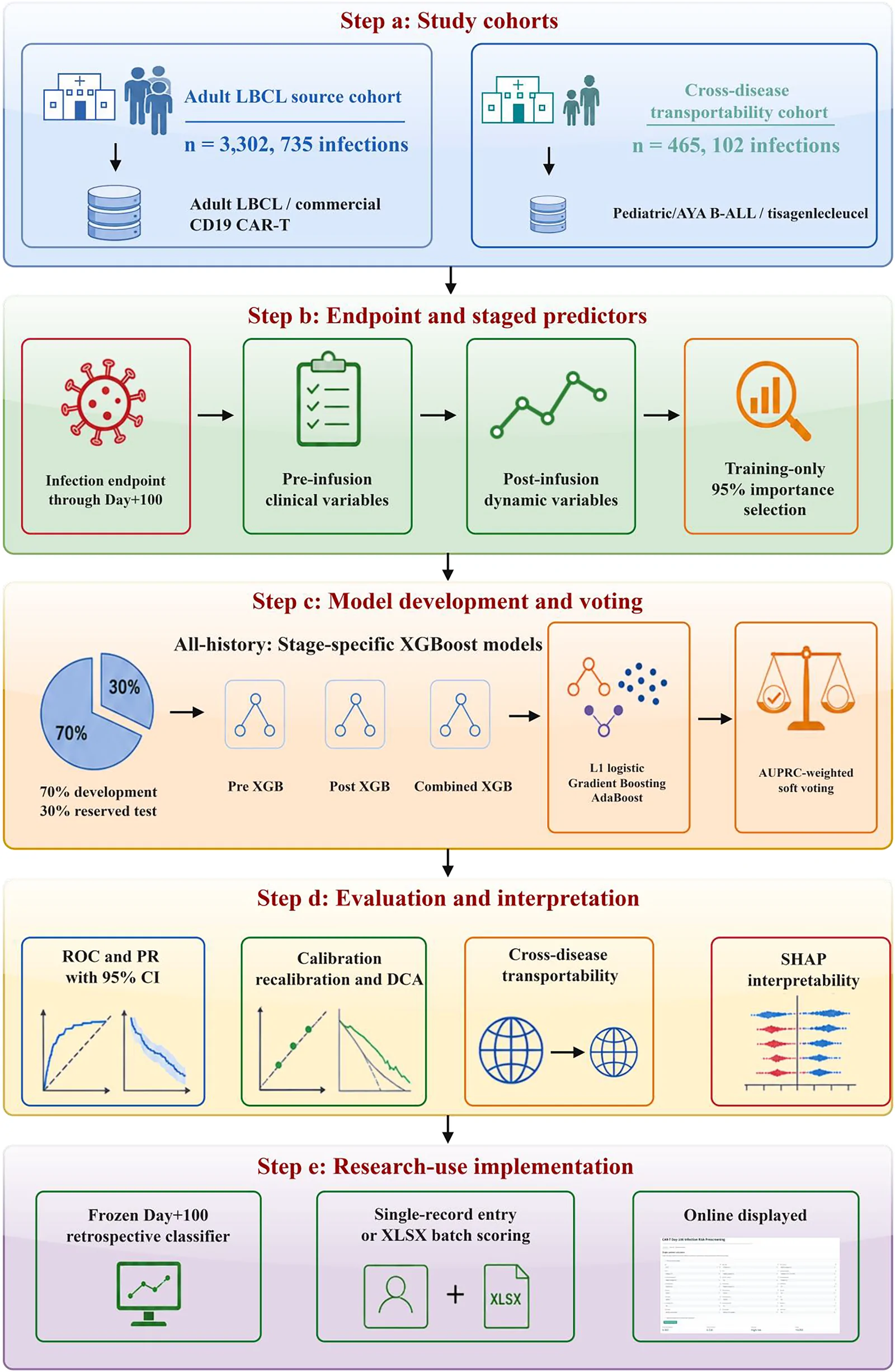
Study cohorts, analysis windows and workflow. **(a)** Adult LBCL and pediatric/AYA B-ALL cohorts. **(b)** Completed-record classification and the corrected Day+14 at-risk analysis, with exclusions and outcome windows. **(c)** All-history model development and the separate Day+14 comparison. **(d)** Performance, calibration, cross-disease assessment and interpretation. **(e)** Implementation of the frozen Day+100 classifier. Model selection and recalibration use development data. Icons are schematic; **Figure 6** shows the running interface.

### Endpoint and observation time

The binary endpoint was the source indicator of infection through Day+100. The source study recorded clinically significant infections requiring treatment (Wudhikarn et al., 2025). Its time-to-first-infection analysis treated relapse or progression, non-infection death and subsequent hematopoietic cell transplantation as competing events. We used the released infection indicator (infecn_new) and its corresponding infection, competing-event or observation-ending time (inctinfec_100). A negative indicator denotes no infection recorded before the source endpoint; observation duration varies by patient. Overall survival status and time were audited separately.

Bacterial, viral and fungal indicators were summarized as patient counts, including overlap. The public file did not distinguish clinically suspected from microbiologically confirmed cases or provide hospitalization, intensive-care admission or a graded infection-severity endpoint. Infection-related mortality was audited as a separate source outcome. These fields and all infection-timing fields were excluded from model inputs. **Supplementary Tables S4** and **S5** document endpoint construction and the available clinical measurements.

### Predictors and temporal definitions

The original all-history candidate set contained 30 clinical variables: 18 pre-infusion and 12 follow-up. Pre-infusion variables included age, performance status, comorbidity, prior infection, prior transplantation, treatment history, CAR-T product, bridging therapy and the interval from lymphodepletion initiation to infusion. Follow-up variables described toxicity, its onset, supportive treatment and hypogammaglobulinemia. Native continuous, ordinal and grouped representations were retained in the original development rule; the clinical label map now identifies bridging therapy and the lymphodepletion-to-infusion interval consistently across tables, figures and the application.

Corticosteroid, tocilizumab and intravenous immunoglobulin (IVIG) exposure, hypogammaglobulinemia, and maximum CRS and ICANS grades lacked dates adequate to establish their order relative to infection. Their grouped grade encodings share that timing limitation. Two additional source status variables, crsstatnew and neutoxnew, were explicitly constructed over an infection-dependent observation window. We evaluated the frozen all-history model and repeated development after removing these two status fields. The Day+14 models used baseline variables and only toxicity occurrence and onset information established by Day+14. A recorded toxicity onset at or before Day+14 defined the corresponding early indicator. Missing onset was coded as no recorded early onset, with the time retained as missing and early toxicity occurrence unresolved.

### Patient partition and landmark eligibility

The original all-history partition was a stratified 70:30 split with random seed 20260603, giving 2,311 development patients with 514 infection events and 991 test patients. The development subset supplied all model and threshold choices. For the revised landmark cohort, eligibility required a valid source endpoint time greater than 14 days. We reconstructed patient membership in the original landmark partition and then removed ineligible patients without drawing a new split. This preserves partition provenance while excluding patients whose infection, competing event or observation ended by Day+14.

The corrected Day+14 development cohort contained 2,014 patients with 262 subsequent infections. The baseline model used 18 clinical variables; the dynamic model used 22, adding CRS and ICANS occurrence and onset known at Day+14. Each used the same sensitivity target of 0.90 for threshold selection. The original baseline target of 0.88 was retained as a historical-rule comparison. A sensitivity analysis excluded endpoint-negative patients recorded alive with overall follow-up shorter than 100 days. Eligibility for this sensitivity analysis used overall-survival follow-up. Source endpoints before Day+100 include competing events whose individual categories are incompletely specified in the released data.

### Preprocessing and model development

The original preprocessing rule treated variables as continuous when at least 85% of values were numeric and more than eight distinct values were present. Other fields, including numeric variables with substantial missingness, were categorical. Each fitting dataset supplied median imputation, standardization, missing categories and one-hot encoding. This produced 554 pre-infusion, 91 follow-up and 148 selected-combined encoded columns in the original adult models. Three XGBoost models represented the respective stages (Chen and Guestrin, 2016). The combined model retained the smallest set reaching 95% cumulative clinical-variable importance, calculated as the average of normalized gain, permutation and mean absolute SHAP importance (Breiman, 2001; Lundberg and Lee, 2017; Lundberg et al., 2020). Supplementary Methods give the fixed grid and model dimensions.

The all-history candidate pool also included L1-penalized logistic regression, gradient boosting and AdaBoost (Tibshirani, 1996; Freund and Schapire, 1997; Friedman, 2001; Pedregosa et al., 2011). Candidate ensembles used simple averaging, AUPRC-weighted averaging, rank averaging or hard voting. Candidate cutoffs and ensemble choices used development out-of-fold (OOF) predictions; the selected all-history model combined six members with AUPRC-proportional weights and a score cutoff of 0.31. This weighted score was evaluated for calibration as described below.


$$s_{i}=\sum_{m=1}^{M}w_{m}{\hat{p}}_{i,m},w_{m}=\frac{{\text{AUPRC}}_{m}}{\sum_{l=1}^{M}{\text{AUPRC}}_{l}}$$

### Validation of the development procedure

The original procedure selected combined-model variables using the development dataset before obtaining selected-model OOF predictions. Five fixed stratified outer folds within the original 70% development subset evaluated the complete development procedure. Each outer training fold repeated preprocessing, importance selection, inner five-fold parameter search, component fitting, ensemble choice and cutoff selection before prediction of the isolated validation fold. Feature selection remained at the outer-training level, as in the original rule. Outer-fold predictions evaluate the full development procedure, whereas inner OOF predictions support selection. We retained the actual scores and classifications of each selected voting strategy and recorded failed selection constraints.

The same outer-fold approach evaluated the corrected landmark development rules. We report individual outer-fold performance, selected variables, weights and thresholds as measures of stability. The reserved test sets evaluate the fitted models. These test sets had already been examined in the original analysis. Supplemental analyses introduced during revision reuse these fixed test patients, with all tuning and recalibration restricted to development data. Bootstrap intervals quantify patient-sampling uncertainty conditional on the fitted models and fixed cutoffs.

### Calibration and decision curves

Calibration was summarized by quantile-bin curves, calibration-in-the-large, the joint logistic calibration intercept and slope, Brier score and log loss (Brier, 1950; Van Calster et al., 2019). A constant prediction equal to the development event rate served as a reference. Logistic recalibration mapped the logit of a clipped model score to an updated probability. The calibrator was fitted to cross-fitted development scores and then frozen for test evaluation. Cross-fitting used the same six-member weighted scoring rule for the primary all-history model; separate scoring systems received separate calibrators. The common-feature calibrator used only adult-cohort scores and outcomes.


$$\text{logit}({\tilde{p}}_{i})=\alpha +\beta \text{logit}(\text{clip}(s_{i},10^{-6},1-10^{-6})).$$

Raw and recalibrated results were compared using the same test patients and paired bootstrap resamples. When comparing the same classification rule, we transformed the original score threshold through the fitted calibration map. Decision curve analysis compared model-guided action with acting for everyone or no one over thresholds 0.01-0.99, retaining negative net benefit. The hypothetical action was additional outpatient monitoring. This exploratory range describes sensitivity to assumed missed-event costs and monitoring burdens; a clinical action threshold requires prospective estimation of those consequences.


$$\text{NB}(p_{t})=\frac{\text{TP}(p_{t})}{n}-\frac{\text{FP}(p_{t})}{n}\frac{p_{t}}{1-p_{t}}.$$

### Additional analyses and model interpretation

A Day+14 neutrophil-recovery sensitivity analysis used the recovery/status fields and recorded times to classify recovery established by Day+14, recovery not established by that date, or insufficient information. Infection-window-truncated recovery fields were excluded. The augmented dynamic model used the same patient partition and development rule. We also report the original redundant-encoding sensitivity analysis and the new removal of the two infection-window toxicity status variables.

SHAP displays describe the frozen combined XGBoost component using all 24 retained input variables, with encoded-column contributions aggregated to the clinical-variable level (Lundberg and Lee, 2017; Lundberg et al., 2020). **Figure 2** shows all 24 variables; **Figure 3** displays the eight largest mean absolute contributions from this complete model. The complete set of dependence plots appears in **Supplementary Figures S2**, **S3**. Display order uses the fixed training sample with stable variable-order tie breaking. Continuous-variable trends are descriptive, and category displays retain their native coding.

**Figure 2:**
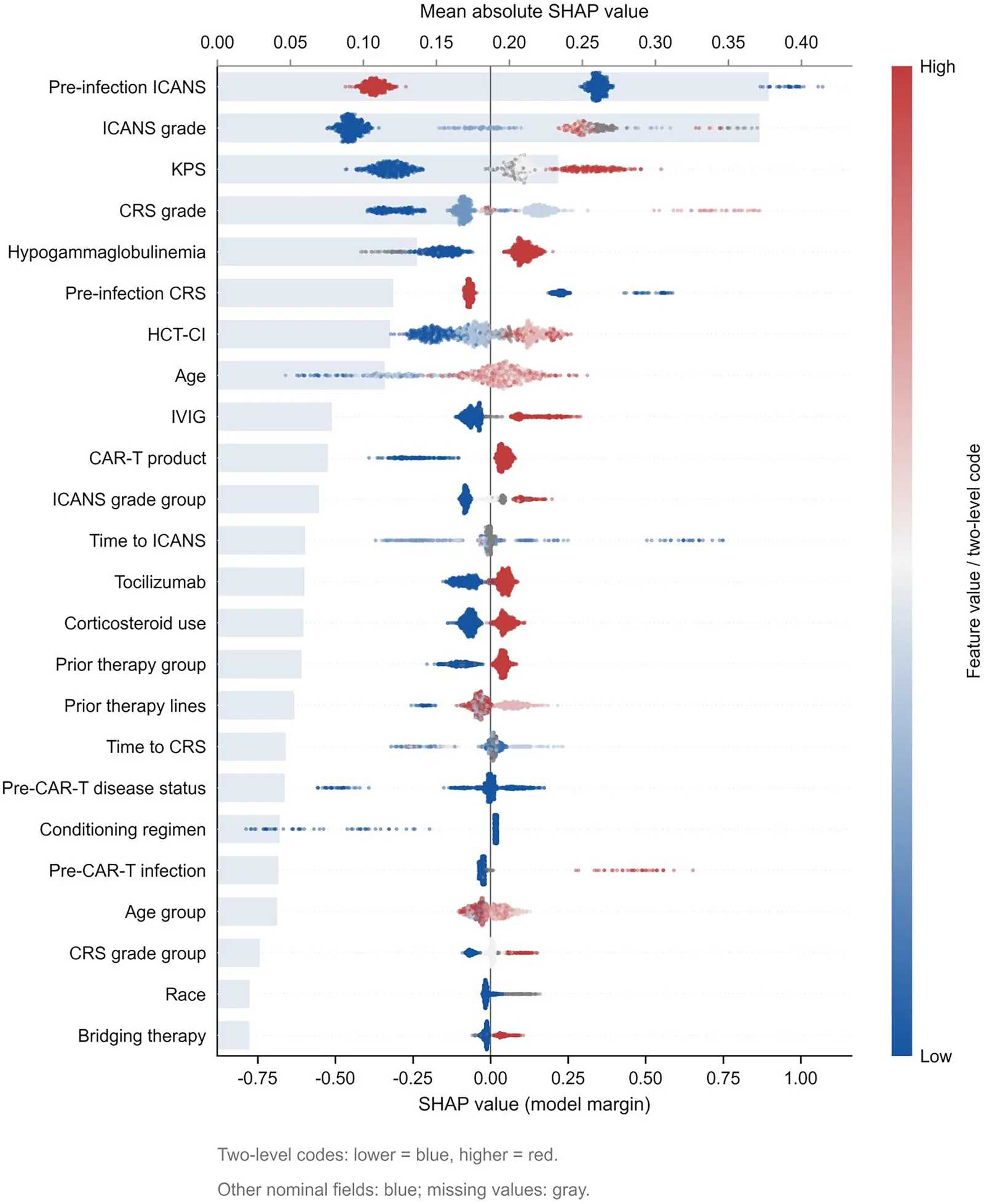
Clinical-variable SHAP interpretation of the frozen combined XGBoost model. Pale horizontal bars show mean absolute contributions on the upper axis; superimposed points show individual contributions on the lower axis for all 24 variables in 1,000 sampled training records (seed 20260603). Encoded-column contributions are summed to each clinical variable on the model margin scale. Numeric and ordered values use the blue-to-red scale, clipped at the 1st and 99th percentiles for color only. Two-level registry codes use blue for the lower code and red for the higher code; other nominal fields are blue and missing values gray. Category colors identify codes, not clinical severity. Vertical displacement separates coincident points. Every sampled record is retained. KPS, Karnofsky performance status; HCT-CI, hematopoietic cell transplantation comorbidity index; IVIG, intravenous immunoglobulin; SHAP, Shapley additive explanations.

**Figure 3:**
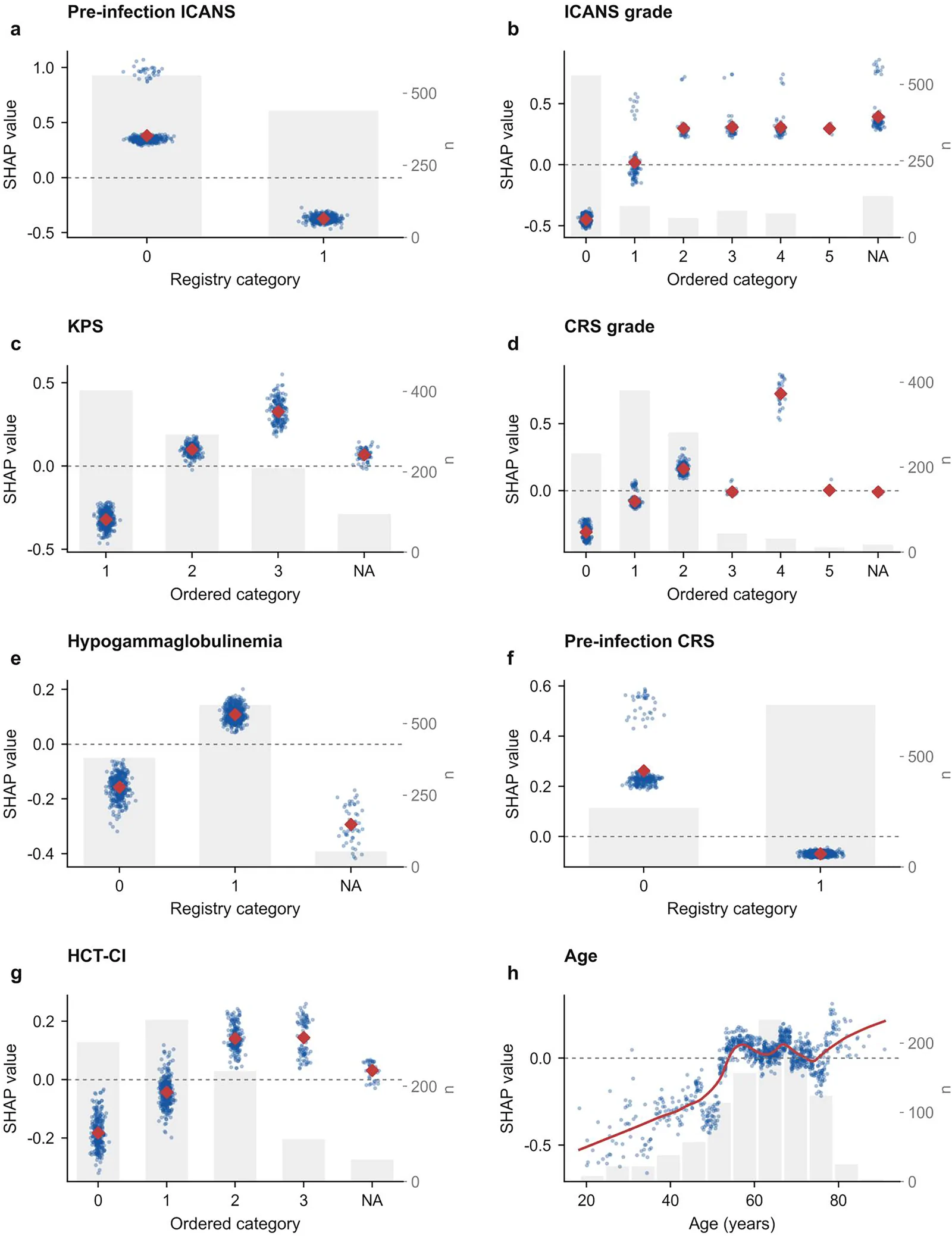
SHAP dependence for the eight leading features among the combined XGBoost model’s 24 input variables. Panels **(a–h)** follow mean absolute SHAP importance in 1,000 training records, with contributions calculated using the complete 24-variable model. Points show individual contributions; red diamonds indicate category means and curves indicate LOWESS trends. Pale gray bars show record counts on the right-hand n axis. NA denotes missing categories. **Supplementary Figures S2**, **S3** show all 24 variables; category mappings and per-panel counts accompany the source data.


$$\Phi_{ig}=\sum_{j\in \text{G}(g)}\varphi_{ij},f(x_{i})=\varphi_{0}+\sum_{g}\Phi_{ig}.$$

### Cross-disease evaluation

The leukemia cohort was selected after comparison for its fit to the cross-disease design: the shared registry framework, Day+100 infection outcome and overlapping clinical fields supported a common-feature evaluation across disease and age settings (Rangarajan et al., 2025; Wudhikarn et al., 2025). The classifier used all 3,302 adult records and 22 common variables: 13 pre-infusion and nine follow-up inputs. The combined branch retained 17 variables; the six-member ensemble also included the two stage-specific models. Adult data supplied feature selection, tuning, the score, cutoff and recalibration. Adult source-cohort OOF results and fixed-model results in 465 leukemia patients are reported separately (**Table 1**; **Supplementary Table S2**).

**Table 1:** Common-feature classifier in the adult LBCL source cohort and pediatric/AYA B-ALL cross-disease evaluation.

Metric	Adult LBCL source cohort (OOF)	Pediatric/AYA B-ALL cross-disease evaluation
Patients	3302	465
Infections	735	102
Score cutoff	0.330	0.330
AUROC	0.714 (0.690–0.736)	0.663 (0.604–0.724)
AUPRC	0.520 (0.482–0.553)	0.356 (0.282–0.454)
Brier score	0.189 (0.185–0.192)	0.172 (0.157–0.187)
Sensitivity	0.893 (0.868–0.914)	0.569 (0.467–0.666)
Specificity	0.257 (0.241–0.275)	0.683 (0.633–0.731)
PPV	0.256 (0.239–0.273)	0.335 (0.265–0.411)
NPV	0.893 (0.869–0.915)	0.849 (0.803–0.888)
Above-cutoff proportion	0.776 (0.761–0.790)	0.372 (0.328–0.418)
Missed infections	79/735	44/102

The 0.33 score cutoff belongs to the separate common-feature soft-mean model. Adult source-cohort OOF estimates use all 3,302 adults; pediatric/AYA B-ALL cross-disease estimates use the 465 evaluation patients. Adult OOF predictions were reconstructed by replay of the original source development procedure; feature selection preceded OOF scoring, so these are selection-stage estimates. Count-based intervals are exact binomial; other intervals are conditional bootstrap. Reconstruction differences from archived discrimination estimates were below 0.000002. Outer procedure-validation results appear separately in **Supplementary Table S6**.

### Statistical reporting

Discrimination was measured by AUROC and AUPRC (Saito and Rehmsmeier, 2015); operating characteristics included sensitivity, specificity, positive and negative predictive values, the proportion above the cutoff and missed infections. Count-based operating characteristics and group event rates used exact binomial 95% confidence intervals; low-score groups also reported size, coverage and infection count. Three score intervals were reported when the fitted low boundary was below 0.50. Discrimination, probability-error and calibration-coefficient percentile bootstrap intervals used 1,000 patient resamples of fixed predictions; paired comparisons used identical resamples. Outer-fold ranges describe procedure stability. Baseline characteristics used standardized mean differences. Analyses used Python with versioned configuration, patient partitions and model metadata.

### Reproducibility implementation

The Streamlit implementation loads the frozen all-history bundle for retrospective classification from completed Day+100 records (Streamlit, n.d.). Single-record and batch outputs identify the model version, score, fixed cutoff and calibration status. The interface is labeled experimental and intended for research users familiar with the clinical data dictionary. Recalibration models are supplied as supplementary analysis versions. The revision package contains the analysis programs, aggregate outputs, figures and deployment materials; patient-level intermediate files remain in the private analysis directory.

## Results

### Cohort and infection outcomes

The adult analysis included 3,302 patients with 735 infection outcomes (22.3%). The corrected Day+14 cohort contained 2,876 patients and 375 infections (13.0%): 2,014 patients with 262 events in development and 862 with 113 events in testing. **Figure 1** shows the cohort flow. **Table 2** describes the adult cohort.

**Table 2:** Selected adult-cohort characteristics by recorded infection status.

Characteristic	Overall	No infection	Infection	SMD
Patients, n	3302	2567	735	
Age, years, median (IQR)	62.5 (53.8-69.4)	62.3 (53.1-69.1)	63.6 (55.0-70.2)	0.14
Prior HCT, yes	850 (25.8)	668 (26.1)	182 (24.8)	0.093
Lower KPS category	634 (21.5)	430 (18.7)	204 (31.0)	0.317
Higher HCT-CI category	293 (9.3)	203 (8.3)	90 (12.9)	0.244
Pre-CAR-T infection, yes	132 (4.2)	87 (3.6)	45 (6.4)	0.129
Hypogammaglobulinemia, yes	1837 (59.6)	1371 (57.2)	466 (67.6)	0.204
Steroid use, yes	1433 (44.3)	1004 (39.9)	429 (59.9)	0.407
Tocilizumab, yes	1789 (54.9)	1318 (52.0)	471 (65.2)	0.273
IVIG, yes	856 (26.9)	607 (24.5)	249 (35.4)	0.236
CRS within the source infection window, yes	2492 (75.5)	1922 (74.9)	570 (77.6)	0.063
ICANS within the source infection window, yes	1410 (42.7)	1076 (41.9)	334 (45.4)	0.071
CRS grade 3-5	262 (7.9)	162 (6.3)	100 (13.6)	0.265
ICANS grade 3-5	569 (17.2)	371 (14.5)	198 (26.9)	0.41

Values are n (%) unless otherwise specified. Denominators exclude missing values for each characteristic. SMD, standardized mean difference; HCT-CI, hematopoietic cell transplantation comorbidity index; KPS, Karnofsky Performance Status. Source-window CRS and ICANS status fields depend on infection ascertainment time.

Bacterial, viral and fungal infections were recorded in 453 (13.7%), 335 (10.1%) and 88 (2.7%) of the 3,302 adults, respectively. Among the 735 infected patients, 608 had one subtype, 113 had two and 14 had all three. These are patient counts, including overlapping subtypes. The separate infection-related mortality field was positive in 51 patients; eight had a negative primary infection indicator. The audit retains this discordance explicitly (**Supplementary Table S4**).

### Completed-record classification

The frozen six-member weighted all-history ensemble had test AUROC 0.724 (95% CI, 0.685–0.765) and AUPRC 0.532 (95% CI, 0.467–0.603) (**Table 3**; **Figure 4**). At score cutoff 0.31, sensitivity was 91.4% (95% CI, 86.9–94.7%), specificity 22.7% (95% CI, 19.8–25.9%), PPV 25.3% (95% CI, 22.4–28.5%) and NPV 90.2% (95% CI, 85.1–94.0%). The cutoff placed 194/991 patients below the boundary, including 19/221 infections. Stage-specific comparisons appear in **Supplementary Figure S1**.

**Table 3:** Frozen all-history ensemble in 991 adult test patients.

Metric	Weighted ensemble
Patients	991
Infections	221
Score cutoff	0.310
AUROC	0.724 (0.685–0.765)
AUPRC	0.532 (0.467–0.603)
Brier score	0.185 (0.178–0.192)
Sensitivity	0.914 (0.869–0.947)
Specificity	0.227 (0.198–0.259)
PPV	0.253 (0.224–0.285)
NPV	0.902 (0.851–0.940)
Above-cutoff proportion	0.804 (0.778–0.829)
Missed infections	19

Count-based proportions use exact binomial 95% intervals; discrimination and probability error use conditional percentile bootstrap intervals. Cutoff-dependent values refer to raw scores. AUROC, area under the receiver operating characteristic curve; AUPRC, area under the precision-recall curve; PPV, positive predictive value; NPV, negative predictive value.

**Figure 4:**
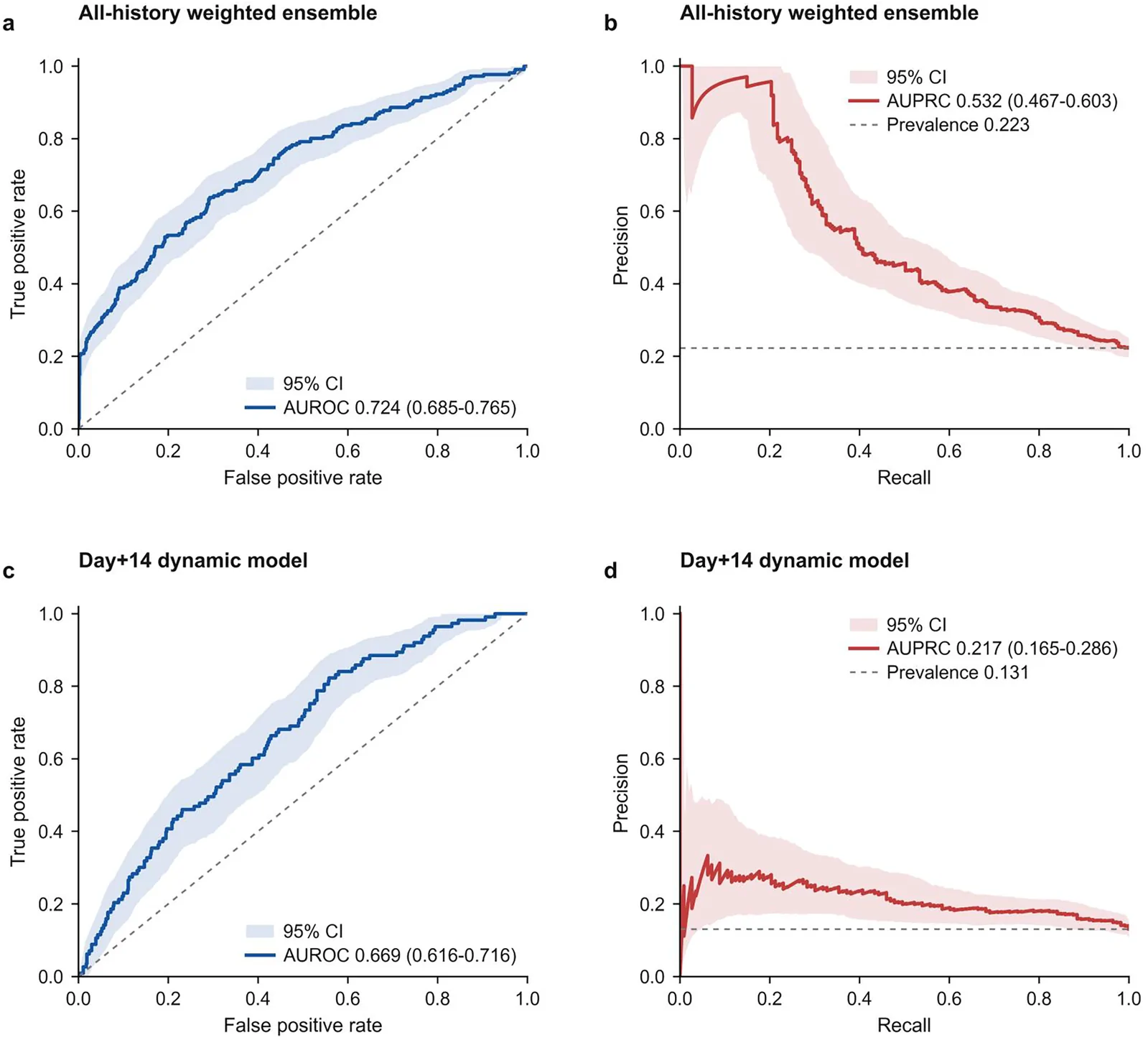
Discrimination in the two held-out analyses. **(a, b)** Receiver operating characteristic (ROC) and precision-recall (PR) curves for the all-history AUPRC-weighted ensemble. **(c, d)** Corresponding curves for the corrected Day+14 dynamic model. All-history weighted ensemble: n=991, 221 events; Day+14 dynamic model: n=862, 113 events. Shaded regions are pointwise 95% percentile bootstrap intervals from 1,000 patient-level resamples. Displayed area intervals are taken from the corresponding locked analysis metrics. Dashed lines indicate chance ROC discrimination and the respective held-out infection prevalence for PR. The two analyses have distinct eligible populations and outcome windows. AUPRC, area under the PR curve; AUROC, area under the ROC curve.

### Day+14 prediction of subsequent infection

In the corrected Day+14 test cohort, 113/862 patients (13.1%) had a subsequent recorded infection. The baseline and dynamic models had AUROCs of 0.650 (95% CI, 0.590–0.701) and 0.669 (95% CI, 0.616–0.716), and AUPRCs of 0.192 and 0.217, respectively. The paired AUROC difference was +0.018 (95% CI, -0.008 to +0.047). Both thresholds were selected with a development sensitivity target of 0.90 (**Table 4**).

**Table 4:** Corrected Day+14 models with the same development sensitivity target of 0.90.

Metric	Baseline	Baseline plus Day+14 toxicity
Patients	862	862
Infections	113	113
Score cutoff	0.100	0.250
AUROC	0.650 (0.590–0.701)	0.669 (0.616–0.716)
AUPRC	0.192 (0.150–0.244)	0.217 (0.165–0.286)
Brier score	0.215 (0.206–0.225)	0.198 (0.188–0.207)
Sensitivity	1.000 (0.968–1.000)	0.956 (0.900–0.985)
Specificity	0.004 (0.001–0.012)	0.207 (0.178–0.238)
PPV	0.132 (0.110–0.156)	0.154 (0.128–0.183)
NPV	1.000 (0.292–1.000)	0.969 (0.929–0.990)
Above-cutoff proportion	0.997 (0.990–0.999)	0.814 (0.787–0.840)
Missed infections	0	5

Count-based proportions use exact binomial 95% intervals; discrimination and probability error use conditional percentile bootstrap intervals. Cutoff-dependent values refer to raw scores. AUROC, area under the receiver operating characteristic curve; AUPRC, area under the precision-recall curve; PPV, positive predictive value; NPV, negative predictive value.

At the dynamic-model score cutoff of 0.25, the low-score group contained 160/862 patients (18.6%) and 5 infections (3.1%; exact 95% CI, 1.0–7.1%). These events represented 4.4% of all test infections. The 702 patients above the cutoff included 108 infections, giving PPV 15.4%. The score intervals yielded: low group, 5/160 infections (3.1%); intermediate group, 55/449 infections (12.2%); high group, 53/253 infections (20.9%) (**Supplementary Table S3**).

### Calibration and decision curves

The original all-history scores exceeded observed infection rates in 8/8 revised quantile groups. Raw Brier score was 0.185, compared with 0.173 for constant development prevalence. Logistic recalibration changed Brier score to 0.144. For the dynamic Day+14 model, raw and recalibrated Brier scores were 0.198 and 0.110, compared with reference 0.114. **Figure 5** and **Supplementary Table S7** report calibration intercepts, slopes and log loss. Original score cutoffs and their mapped probability cutoffs preserve the same classifications.

**Figure 5:**
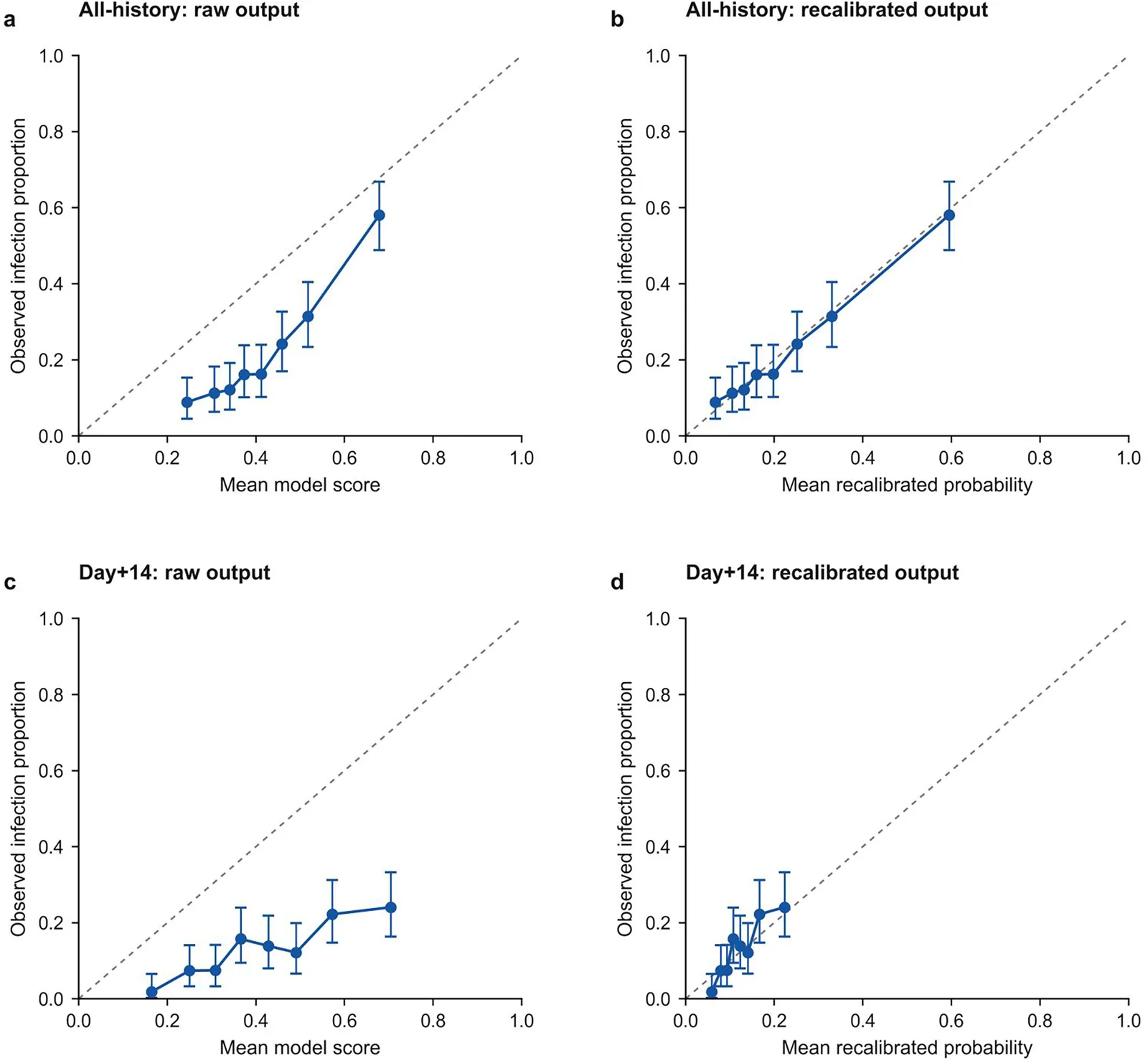
Held-out calibration before and after development-only recalibration. **(a, b)** All-history weighted ensemble. **(c, d)** Corrected Day+14 dynamic model. Points compare mean raw model score or recalibrated probability, as applicable, with the observed infection proportion in eight quantile groups; vertical bars are 95% exact binomial intervals. Fewer groups are used if tied scores prevent distinct groups. Recalibration is estimated exclusively from development predictions and applied unchanged to the held-out observations. Dashed lines indicate equality between model output and observed event proportion. Bin counts and events are supplied in the figure source data.

Decision curves retained the full explored range and negative values (**Supplementary Figure S5**). All-history raw net benefit was -0.066 at threshold 0.31; Day+14 raw net benefit was -0.104 at threshold 0.25; cross-disease common-feature raw net benefit was 0.003 at threshold 0.33. These raw-score calculations expose the effect of treating uncalibrated scores as action probabilities. Recalibrated curves and both reference strategies are shown on the same patients, with numerical values in the aggregate workbook.

### analyse Procedure stability and sensitivity s

Outer-fold AUROC ranged from 0.651–0.728 for the all-history development procedure, 0.562–0.670 for the baseline landmark procedure and 0.572–0.687 for the dynamic procedure. Their AUPRC ranges were 0.436–0.557, 0.160–0.274 and 0.169–0.324. **Supplementary Table S6** reports fold performance and unmet development-selection constraints. Selected strategies, variables and cutoff stability are provided in Revision_results.xlsx and analysis/outer_fold_metrics.csv.

Removing the two infection-window-derived CRS/ICANS status fields produced all-history test AUROC 0.727 (95% CI, 0.687–0.767) and AUPRC 0.535 (95% CI, 0.469–0.604); the paired AUROC change was +0.002 (95% CI, -0.004 to +0.010). The Day+14 recovery audit classified 2,292 patients as recovered by Day+14, 566 as not recovered by Day+14 and 18 as having insufficient information. Adding recovery status gave dynamic-model AUROC 0.674, with paired change +0.005 (95% CI, -0.012 to +0.020). Excluding the 84 landmark endpoint-negative patients recorded alive with follow-up under 100 days gave dynamic-model AUROC 0.684. **Supplementary Table S8** summarizes sensitivity results; analysis/metrics.csv (Revision_results.xlsx, Metrics) includes complete metrics and baseline follow-up exclusion.

### Cross-disease evaluation

The leukemia cohort had 102/465 infection outcomes (21.9%) and differed from the adult lymphoma cohort in age, disease and CAR-T product (**Supplementary Table S2**). The common-feature model had adult source-cohort OOF AUROC 0.714 and cross-disease AUROC 0.663 (95% CI, 0.604–0.724). At its fixed score cutoff of 0.33, B-ALL sensitivity was 56.9%, specificity 68.3%, PPV 33.5% and NPV 84.9%; 44/102 infections lay below the cutoff (**Table 1**). Source-trained recalibration changed B-ALL Brier score from 0.1723 to 0.1722, while log loss increased from 0.5277 to 0.5456 (**Supplementary Figure S4**; **Supplementary Table S7**). Thus the source-trained map did not consistently improve cross-disease probability error.

### Clinical-variable interpretation and implementation

The frozen combined XGBoost component retained 24 clinical variables using the cumulative-importance rule (**Supplementary Table S1**). **Figure 2** shows the clinical-variable SHAP distribution, and **Figure 3** shows the eight leading variables in a two-column, four-row layout. All 24 dependence plots are shown over two supplementary pages. The fixed SHAP sample, ranking and clinical labels are shared across these displays. The application reproduces the frozen weighted ensemble, while these explanations refer to its combined XGBoost component.

The revised single-record and batch interfaces display the score, classification cutoff, model version and calibration status together (**Figure 6**). Model files retain their original identifiers and numerical scoring. The local deployment package contains the revised interface and reproducibility instructions.

**Figure 6:**
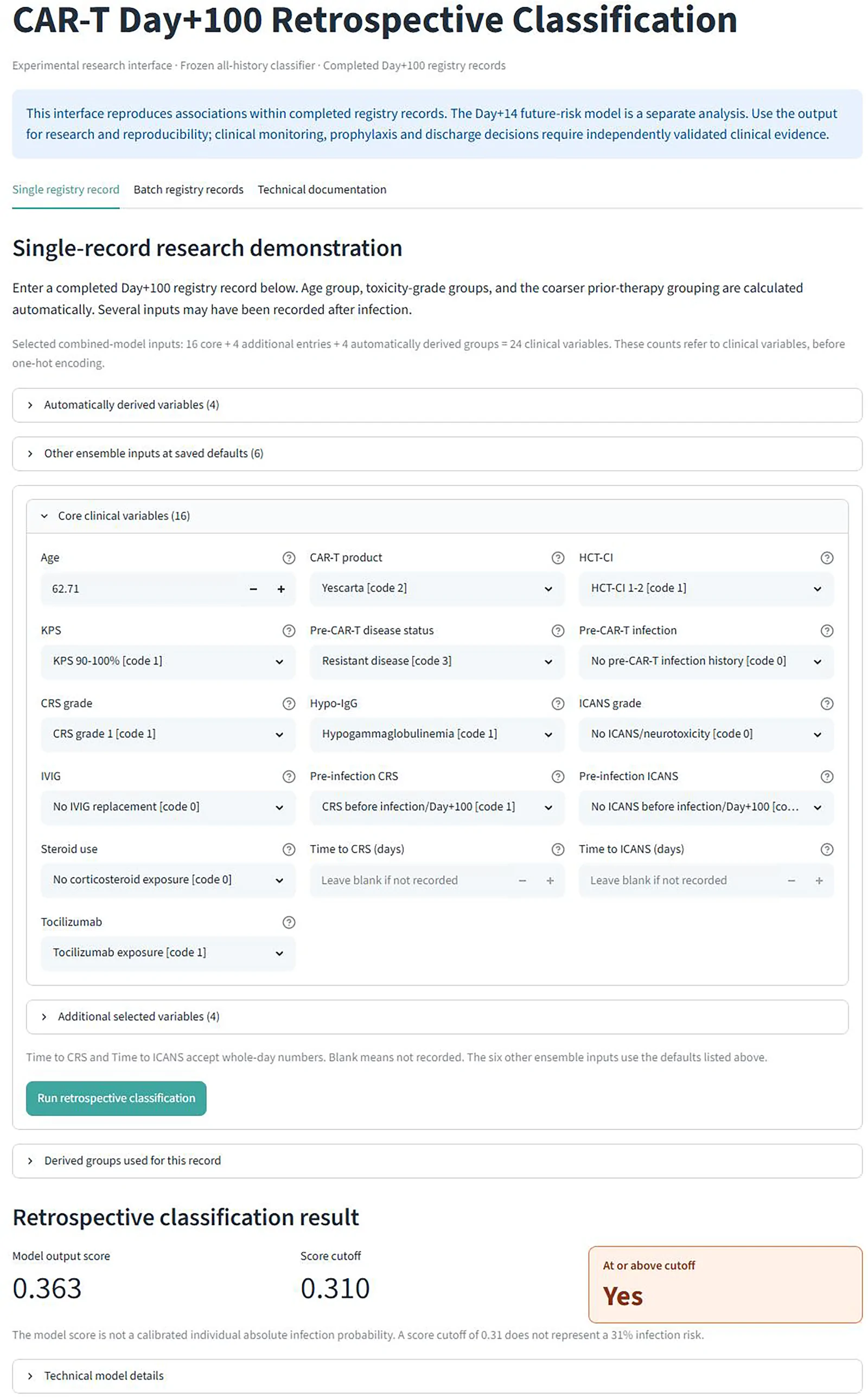
Revised experimental interface for retrospective Day+100 classification. The result view identifies the frozen model score, threshold and calibration status. Single-record and batch outputs use the same definitions. The revised research-use interface is available at https://car-t-infection-risk-calculator.streamlit.app/.

## Discussion

The two analysis branches capture different information about infection after CAR-T therapy. Completed records provided all-history discrimination of AUROC 0.724; Day+14 information provided moderate discrimination for later recorded infection (AUROC 0.669). The Day+14 low-score group covered 18.6% of test patients and contained 5/113 infections. Discrimination, calibration and subgroup event counts together characterize the information available for planning monitoring studies.

The low-score group provides a defined population for prospective comparison of outpatient monitoring schedules. Such studies would measure infections, delayed recognition, unscheduled care, patient burden and resource use. Group size determines recruitment potential, and its observed event rate informs monitoring requirements. Decision curves describe tradeoffs under assumed monitoring burdens and missed-event costs; the effects of changing surveillance require prospective measurement. Monitoring studies would operate within established prevention and treatment protocols (Hill and Seo, 2020; Shahid et al., 2024).

Toxicity and supportive-treatment variables describe the post-infusion course. Corticosteroid and tocilizumab exposure can reflect CRS or ICANS severity and treatment; IVIG use can reflect immune dysfunction and treatment decisions. SHAP contributions describe associations shaped by these clinical indications and treatment patterns, rather than causal drug effects. The removal analysis quantifies the influence of toxicity status fields whose ascertainment window was defined by infection.

The transfer assessment quantified performance across an adult lymphoma to pediatric/young-adult leukemia setting. Its fixed cutoff missed 44/102 infections, and source-trained recalibration changed cross-disease probability error as reported above. Differences in disease, age, product and predictor availability can alter both ranking and the score-to-event relationship. Independent adult-LBCL evaluation is the next step for assessing target-population generalizability before testing a monitoring strategy.

The public registry supports a multicenter analysis of recorded infection within the source observation and competing-event framework. Missing toxicity-treatment dates, longitudinal lymphocyte/B-cell recovery and immunoglobulin measurements, detailed antimicrobial prophylaxis and CMV/EBV surveillance, baseline nutrition and timed post-CAR-T disease response limit temporal interpretation and adjustment (**Supplementary Table S5**). Prospective data collection should capture these measurements alongside infection outcomes. Neutrophil-recovery timing and categorical hypogammaglobulinemia provide partial measures of immune recovery and immunoglobulin deficiency; longitudinal laboratory data would characterize their trajectories.

Model-selection uncertainty was evaluated by repeating the development procedure within isolated outer folds. Conditional test-set bootstrap intervals quantify patient-sampling variability for a fitted model and fixed cutoff, while fold-level variation describes instability in the development rule. Center-level performance variation and demographic fairness require dedicated evaluation before clinical use.

## Conclusion

Completed Day+100 records identify combinations of toxicity, baseline health and supportive-care features associated with recorded infection. The corrected Day+14 analysis measures subsequent-event discrimination, calibration and the coverage of a low-score subgroup. Prospective adult-lymphoma studies can now evaluate whether that subgroup supports different outpatient monitoring schedules with acceptable event detection and patient burden.

## Data Availability

The original contributions presented in the study are included in the article/**Supplementary Material**. Further inquiries can be directed to the corresponding authors.
