# Supplementary material for: Temporal clinical trajectories and Day+14 landmark stratification of infection risk after CD19 CAR T-cell therapy
Source: Front Cell Infect Microbiol. 2026 Oct 23;16:1933555. doi: 10.3389/fcimb.2026.1933555 (PMC13739542; doi:10.3389/fcimb.2026.1933555)
Supplement: Supplementary file 1 [file SupplementaryFile1.docx]

Supplementary methods and results

Temporal Clinical Trajectories and Day+14 Landmark Stratification of Infection Risk after CD19 CAR T-Cell Therapy

This supplement documents source definitions, development rules, stability, calibration and sensitivity analyses.

# Supplementary Methods

## Data and preprocessing audit

The adult source workbook contains one non-patient descriptive row, 3,332 numeric patient identifiers and 30 unusable infection indicators. The analytic outcome is the released infecn_new field. Landmark eligibility additionally requires inctinfec_100 >14. Private manifests retain source identifiers, original split membership, endpoint time, survival status and exclusion reasons; aggregate flow appears in Table S4.

The original preprocessing rule classified a feature as continuous when at least 85% of values could be represented numerically and more than eight distinct nonmissing values were present. Other features were treated as categorical, including numeric fields with substantial missingness. Continuous features received median imputation and standardization; categorical features received a missing label and one-hot encoding with unseen categories ignored. Each outer training fold re-estimated this preprocessing. The rule was retained in the revision to evaluate the existing development procedure.

Development sample and encoded model dimensions

| **Branch** | **Patients / events** | **Clinical inputs** | **Encoded columns** |
| --- | --- | --- | --- |
| All-history pre-infusion | 2,311 / 514 | 18 | 554 |
| All-history follow-up | 2,311 / 514 | 12 | 91 |
| All-history combined selected | 2,311 / 514 | 24 | 148 |
| Day+14 baseline | 2,014 / 262 | 18 | 513 |
| Day+14 dynamic | 2,014 / 262 | 22 | 547 |
| Day+14 plus recovery | 2,014 / 262 | 23 | 550 |

The study used all eligible released records. Descriptive event-to-clinical-variable ratios were 17.1 for all-history development (514/30), 14.6 for the Day+14 baseline model (262/18) and 11.9 for the dynamic model (262/22). These ratios summarize initial clinical-variable counts; encoded dimensions are reported separately, and fitted tree complexity also depends on tree structure and selection. Outer-fold validation evaluates the development procedure at this available sample size.

## Parameter search and importance selection

Each XGBoost search compared three fixed parameter sets using five stratified inner folds. Class weighting used the negative-to-positive ratio of the relevant training data. XGBoost parameter ranking used OOF AUPRC followed by AUROC. The random seed was 20260603. Each revision training task also fixed the Python and NumPy random generators to make the original L1 logistic estimator, whose random_state was unspecified, reproducible. Two independent uncached whole-ensemble replays produced identical scores. The fixed test identities were preserved in every sensitivity analysis, with exclusions applied within each original partition.

XGBoost parameter grid retained from the original analysis

| **Parameter** | **Set 1** | **Set 2** | **Set 3** |
| --- | --- | --- | --- |
| Trees | 250 | 350 | 220 |
| Maximum depth | 2 | 2 | 3 |
| Learning rate | 0.04 | 0.03 | 0.04 |
| Subsample | 0.85 | 0.90 | 0.80 |
| Column subsample | 0.85 | 0.90 | 0.80 |
| Minimum child weight | 5 | 3 | 8 |
| L2 penalty | 1 | 2 | 3 |

The other member settings were retained: L1 logistic regression used liblinear, C=0.5, balanced class weights and up to 5,000 iterations; gradient boosting used 180 trees, learning rate 0.05, depth 2, minimum leaf size 40 and subsample 0.85; AdaBoost used 320 decision stumps, learning rate 0.05 and minimum leaf size 30. All members applied their development-fitted preprocessing.

The combined-feature selection rule grouped encoded columns under their original clinical variable. XGBoost gain, permutation importance and mean absolute SHAP importance were separately normalized and averaged. Variables were ranked by that mean, and the smallest cumulative set reaching 95% was retained. Selection used the full relevant development or outer-training subset; the selected-variable inner OOF predictions were used for subsequent choices. The outer validation fold supplied independent performance evaluation.

## Ensemble and threshold rules

The six candidate members were pre-infusion, follow-up and selected-combined XGBoost, plus L1 logistic regression, gradient boosting and AdaBoost on the selected combined variables. Candidate ensembles comprised all-member and top-member soft averages, AUPRC-weighted averages, normalized-rank averages and hard votes. Eligible ensemble candidates were ranked by AUPRC, AUROC, NPV, specificity and then lower above-cutoff proportion. The code archive records the exact members, parameters, candidate scores and constraints for every fold. The main frozen model uses the six-member AUPRC-weighted average; its weights sum to one.

Thresholds were searched from 0.03 to 0.95 in increments of 0.01. The selection utility was 0.30×AUPRC + 0.20×AUROC + 0.25×sensitivity + 0.15×NPV + 0.10×specificity − 0.08×above-cutoff proportion. The original all-history search considered sensitivity targets 0.88, 0.90 and 0.92. Ensemble eligibility required OOF sensitivity ≥0.88, NPV ≥0.88 and above-cutoff proportion ≤0.82; the existing fallback rule was retained when those constraints could not be met. Both corrected landmark models used target 0.90. The baseline target 0.88 was also evaluated as a historical-rule comparison.

Scores and classifications were stored directly for the chosen candidate. A hard-vote candidate retains its component voting thresholds and vote rule; a rank candidate retains its rank scoring rule. Reproduction therefore uses the stored scoring and voting rules. Supplemental cross-fitting for recalibration separately retained the fixed six-member weighted score for the all-history model and fixed soft-mean score for the common-feature model, even when a different strategy won outer-fold selection.

## Calibration estimation and uncertainty

For logistic recalibration, each score was clipped to [10⁻⁶, 1−10⁻⁶] before the logit transform. A binomial logistic mapping with an intercept and slope was fitted to outer cross-fitted development scores without class weighting. The frozen mapping was then applied to the designated evaluation cohort. A mapped cutoff reproduces the original classification because all fitted calibration slopes were positive. Calibration-in-the-large used a fixed slope of one; the jointly fitted intercept and slope were reported separately. Eight quantile bins were used for revised calibration curves, and the original ten-bin all-history analysis was independently audited.

For each fixed score vector, 1,000 patient bootstrap samples generated percentile intervals for discrimination, probability error and calibration coefficients. Paired score comparisons used the same sampled patient identities. Count-based sensitivity, specificity, predictive values, coverage, calibration-bin event rates and low-score event rates used exact Clopper–Pearson binomial intervals. These intervals are conditional on fitted models, selected thresholds and calibration maps. Five outer folds provide procedure-validation estimates, summarized by their observed performance range.

## Decision curve analysis

At an assumed action threshold, net benefit equals the true-positive fraction minus the false-positive fraction multiplied by threshold/(1−threshold). The analysis evaluated 0.01-0.99 by 0.01, with hypothetical additional outpatient monitoring as the action. The threshold represents the assumed relative burden of unnecessary monitoring and missed infection. Raw and recalibrated scores were each compared with acting for everyone and acting for no one. Full negative values were retained. The threshold grid evaluates sensitivity to these assumed burdens and costs.

## Neutrophil recovery and follow-up sensitivity

Source engrafstat denotes recovery or a terminal recovery status; intctengraf records the corresponding time and intctengrafy provides event-only recovery timing. Recovered by Day+14 required engrafstat=1 and 0<intctengrafy≤14. Not recovered by Day+14 comprised status 1 with recovery time >14, or status 0/2 with intctengraf>14. All other records were classified as unknown. Because the eventual status combines recovered and never-dropped counts, establishing recovery at Day+14 required a recorded time. Infection-window-truncated engrafnew and grouped post-hoc times were excluded.

The incomplete-follow-up analysis excluded endpoint-negative patients with dead=0 and inctsurv<100: 85 in the full adult cohort and 84 in the corrected landmark cohort. The source endpoint may end before 100 days for competing events even when overall survival follow-up continues. The sensitivity analysis therefore targets incomplete follow-up among patients recorded alive.

## Reporting records

Methods and versioned code document the analytical procedures for this secondary analysis. Individual outcome and predictor assessor characteristics and blinding procedures are unavailable in the public-use files. Available project records provide no separate prospective protocol, registration identifier or documentation of patient or public involvement in the design of this secondary analysis. Source-study consent and ethics approval are reported in the main Ethics statement.

# Supplementary Tables

Table S1. Frozen combined-model cumulative importance selection.

| **Clinical variable** | **Gain** | **Permutation** | **SHAP** | **Combined** | **Cumulative** | **Retained** |
| --- | --- | --- | --- | --- | --- | --- |
| ICANS grade | 0.066 | 0.265 | 0.149 | 0.160 | 0.160 | Yes |
| ICANS within the source infection window | 0.069 | 0.248 | 0.121 | 0.146 | 0.306 | Yes |
| CRS grade | 0.056 | 0.068 | 0.088 | 0.071 | 0.377 | Yes |
| Time to ICANS | 0.139 | 0.035 | 0.038 | 0.071 | 0.447 | Yes |
| KPS | 0.046 | 0.068 | 0.097 | 0.070 | 0.517 | Yes |
| CRS within the source infection window | 0.030 | 0.065 | 0.041 | 0.045 | 0.563 | Yes |
| HCT-CI | 0.036 | 0.036 | 0.060 | 0.044 | 0.607 | Yes |
| ICANS grade group | 0.065 | 0.007 | 0.031 | 0.034 | 0.641 | Yes |
| Time to CRS | 0.058 | 0.014 | 0.028 | 0.033 | 0.675 | Yes |
| Hypo-IgG | 0.028 | 0.024 | 0.048 | 0.033 | 0.708 | Yes |
| Age | 0.008 | 0.044 | 0.037 | 0.029 | 0.737 | Yes |
| Age group | 0.032 | 0.014 | 0.020 | 0.022 | 0.759 | Yes |
| CRS grade group | 0.048 | 0.004 | 0.014 | 0.022 | 0.781 | Yes |
| CAR-T product | 0.021 | 0.017 | 0.027 | 0.022 | 0.803 | Yes |
| IVIG | 0.022 | 0.015 | 0.028 | 0.022 | 0.825 | Yes |
| Pre-CAR-T disease status | 0.033 | 0.010 | 0.020 | 0.021 | 0.846 | Yes |
| Steroid use | 0.026 | 0.003 | 0.018 | 0.016 | 0.861 | Yes |
| Prior therapy group | 0.018 | 0.006 | 0.022 | 0.015 | 0.877 | Yes |
| Prior therapy lines | 0.019 | 0.009 | 0.016 | 0.015 | 0.891 | Yes |
| Pre-CAR-T infection | 0.018 | 0.012 | 0.014 | 0.015 | 0.906 | Yes |
| Tocilizumab | 0.020 | 0.003 | 0.018 | 0.014 | 0.920 | Yes |
| Conditioning regimen | 0.020 | 0.006 | 0.015 | 0.013 | 0.933 | Yes |
| Race | 0.027 | 0.005 | 0.007 | 0.013 | 0.946 | Yes |
| Bridging therapy | 0.027 | 0.002 | 0.006 | 0.011 | 0.957 | Yes |
| CAR-T dose | 0.017 | 0.006 | 0.010 | 0.011 | 0.969 | No |
| Lymphodepletion-to-infusion interval | 0.008 | 0.008 | 0.009 | 0.008 | 0.977 | No |
| Prior HCT | 0.013 | 0.004 | 0.007 | 0.008 | 0.985 | No |
| Lymphoma transformation | 0.017 | 0.003 | 0.004 | 0.008 | 0.993 | No |
| HCT-to-CAR-T interval | 0.007 | 0.002 | 0.006 | 0.005 | 0.998 | No |
| Disease subtype | 0.006 | 0.000 | 0.001 | 0.002 | 1.000 | No |

Gain, permutation and SHAP columns are normalized within method. Combined importance is their average; cumulative importance determines the 95% retained set.

Table S2. Source-cohort characteristics and infection outcomes.

| **Characteristic** | **Adult LBCL** | **Pediatric/young-adult B-ALL** |
| --- | --- | --- |
| Patients | 3302 | 465 |
| Infections, n (%) | 735 (22.3) | 102 (21.9) |
| Age, years, median (IQR) | 62.5 (53.8-69.4) | 13.8 (7.8-19.3) |
| Age observed, n | 3302 | 465 |
| Tisagenlecleucel, n (%) | 538 (16.3) | 465 (100.0) |
| Axicabtagene ciloleucel, n (%) | 2764 (83.7) | 0 (0.0) |

Age and adult product counts use the current analytic records; the source B-ALL cohort comprised tisagenlecleucel recipients [29]. These public analysis subsets differ from the full cohorts in the source publications. IQR, interquartile range.

Table S3. Corrected Day+14 score groups in the 862-patient test set.

| **Model** | **Score group** | **Patients** | **Infections** | **Coverage** | **Event rate (95% CI)** |
| --- | --- | --- | --- | --- | --- |
| Baseline | low | 3 | 0 | 0.3% | 0.0% (0.0–70.8) |
| Baseline | intermediate | 545 | 49 | 63.2% | 9.0% (6.7–11.7) |
| Baseline | high | 314 | 64 | 36.4% | 20.4% (16.1–25.3) |
| Dynamic | low | 160 | 5 | 18.6% | 3.1% (1.0–7.1) |
| Dynamic | intermediate | 449 | 55 | 52.1% | 12.2% (9.4–15.6) |
| Dynamic | high | 253 | 53 | 29.4% | 20.9% (16.1–26.5) |

The low boundary is 0.10 for the baseline model and 0.25 for the dynamic model; the upper descriptive boundary is 0.50. Both low boundaries use development sensitivity target 0.90. The baseline 0.88 target also selected 0.10 after cohort correction.

Table S4. Source endpoint and cohort audit.

| **Audit item** | **Count** | **Interpretation** |
| --- | --- | --- |
| Numeric source patients | 3,332 | One additional row contains variable descriptions |
| Usable adult infection endpoint | 3,302 | 30 endpoint exclusions |
| Any infection | 735 | Patients, not episodes |
| Bacterial / viral / fungal | 453 / 335 / 88 | Overlapping patient indicators |
| One / two / three subtypes | 608 / 113 / 14 | Among 735 infected patients |
| Infection-related mortality indicator | 51 | Separate endpoint |
| Mortality-positive / infection-negative | 8 | Retained as source discordance |
| Original / corrected landmark | 2,942 / 2,876 | 66 early source endpoints excluded |
| Dead by Day+14 among exclusions | 32 | Death is not an eligible landmark observation |
| Corrected infections | 375 | 262 development and 113 test events |

Table S5. Availability of requested clinical variables and endpoint detail.

| **Clinical item** | **Available evidence** | **Analysis use** |
| --- | --- | --- |
| Neutrophil recovery | Recovery/status and corresponding timing | Day+14 three-state sensitivity |
| Serial absolute neutrophil counts | Not provided | Recovery timing is a partial proxy |
| Lymphocyte and B-cell recovery | Not provided | Prospective data requirement |
| Immunoglobulin trajectory | Categorical hypogammaglobulinemia only | All-history proxy; timing insufficient for Day+14 |
| Antimicrobial prophylaxis | Not provided | No prophylaxis adjustment |
| CMV/EBV surveillance | Monitoring schedule/intensity not provided | No surveillance-practice adjustment |
| Baseline nutrition | Not provided | No nutritional-status adjustment |
| Post-CAR-T disease response | Not provided as a timed predictor | Baseline disease status retained |
| Microbiological confirmation | Separate confirmation flag not provided | Source treatment-requiring infection endpoint |
| Hospitalization / ICU / infection grade | Not provided | Severe-infection model cannot be constructed |
| Infection-related death | Separate IRM_new with discordant records | Endpoint audit, excluded from predictors |
| Bridging therapy | ct_treatbg | Preserved key treatment_background |
| Lymphodepletion-to-infusion interval | timelym | Preserved key disease_interval |
| Conditioning regimen | lymlist | Preserved key lymphoma_disease_list |

Table S6. Outer validation of complete development rules.

| **Procedure** | **Fold** | **AUROC** | **AUPRC** | **Cutoff** | **Above cutoff** | **Constraints met** |
| --- | --- | --- | --- | --- | --- | --- |
| All-history | 1 | 0.728 | 0.557 | 0.11 | 100.0% | False |
| All-history | 2 | 0.688 | 0.529 | 0.32 | 78.1% | True |
| All-history | 3 | 0.705 | 0.514 | 0.31 | 79.2% | True |
| All-history | 4 | 0.651 | 0.436 | 0.29 | 83.3% | True |
| All-history | 5 | 0.727 | 0.537 | 0.33 | 72.7% | True |
| Day14 baseline | 1 | 0.616 | 0.271 | 0.19 | 93.5% | True |
| Day14 baseline | 2 | 0.611 | 0.163 | 0.21 | 91.8% | True |
| Day14 baseline | 3 | 0.562 | 0.164 | 0.28 | 78.7% | True |
| Day14 baseline | 4 | 0.670 | 0.274 | 0.04 | 100.0% | True |
| Day14 baseline | 5 | 0.581 | 0.160 | 0.27 | 85.1% | True |
| Day14 dynamic | 1 | 0.625 | 0.281 | 0.08 | 99.8% | True |
| Day14 dynamic | 2 | 0.604 | 0.179 | 0.20 | 93.8% | True |
| Day14 dynamic | 3 | 0.572 | 0.169 | 0.25 | 84.4% | True |
| Day14 dynamic | 4 | 0.687 | 0.324 | 0.05 | 100.0% | True |
| Day14 dynamic | 5 | 0.601 | 0.179 | 0.18 | 92.8% | True |
| Common | 1 | 0.684 | 0.456 | 0.32 | 79.6% | True |
| Common | 2 | 0.681 | 0.494 | 0.29 | 79.4% | True |
| Common | 3 | 0.693 | 0.505 | 0.31 | 77.7% | True |
| Common | 4 | 0.762 | 0.600 | 0.26 | 75.9% | True |
| Common | 5 | 0.724 | 0.557 | 0.26 | 78.8% | True |

One outer fold failed the original ensemble constraints. The fallback was retained and reported. Constraints met refers to development-side ensemble selection; Above cutoff reports the outer validation patients and need not meet the development constraint. Table S6 summarizes performance and constraint status for each outer fold. The common-feature rows evaluate adult source development; they are separate from B-ALL evaluation performance.

Table S7. Calibration and probability error before and after source-only recalibration.

| **Model** | **Score** | **Cutoff** | **Intercept** | **Slope** | **CITL** | **Brier** | **Log loss** |
| --- | --- | --- | --- | --- | --- | --- | --- |
| All-history | raw | 0.3100 | -0.866 | 1.642 | -0.980 | 0.1853 | 0.5570 |
| All-history | recalibrated | 0.1074 | -0.029 | 1.016 | -0.046 | 0.1440 | 0.4574 |
| Day14 baseline | raw | 0.1000 | -1.783 | 0.805 | -1.784 | 0.2154 | 0.6189 |
| Day14 baseline | recalibrated | 0.0484 | 0.937 | 1.507 | 0.008 | 0.1111 | 0.3751 |
| Day14 dynamic | raw | 0.2500 | -1.701 | 0.796 | -1.691 | 0.1975 | 0.5790 |
| Day14 dynamic | recalibrated | 0.0795 | 0.660 | 1.321 | 0.063 | 0.1098 | 0.3696 |
| Cross-disease common | raw | 0.3300 | -0.473 | 1.144 | -0.567 | 0.1723 | 0.5277 |
| Cross-disease common | recalibrated | 0.1082 | 0.058 | 0.636 | 0.776 | 0.1722 | 0.5456 |

CITL, calibration-in-the-large with slope fixed at one. Intercept and slope are jointly estimated on the evaluation patients as calibration diagnostics. The mapping itself was trained only on development cross-fitted scores. The Brier reference is the constant development prevalence, not the evaluation prevalence. Mapped cutoffs preserve raw-score classifications.

Table S7 continued. Paired changes in Brier score and development-fitted mapping.

| **Model** | **Mapping intercept** | **Mapping slope** | **Calibration n** | **Brier change (95% CI)** |
| --- | --- | --- | --- | --- |
| allhistory original | -0.8242 | 1.6165 | 2311 | -0.041 (95% CI, -0.050 to -0.032) |
| day14 baseline | -1.8049 | 0.5345 | 2014 | -0.104 (95% CI, -0.123 to -0.088) |
| day14 dynamic | -1.7870 | 0.6028 | 2014 | -0.088 (95% CI, -0.106 to -0.073) |
| cross-disease common | -0.8345 | 1.8001 | 3302 | -0.000 (95% CI, -0.016 to +0.016) |

Revised calibration figures use eight quantile bins. Metric intervals, log loss and reference Brier scores are reported in Table S7.

Table S8. Supplemental analyses on preserved test identities.

A. Corrected Day+14 dynamic-model sensitivity analyses

| **Metric** | **Dynamic** | **Plus recovery** | **Follow-up exclusion** |
| --- | --- | --- | --- |
| Test patients | 862 | 862 | 838 |
| Test infections | 113 | 113 | 113 |
| Score cutoff | 0.250 | 0.110 | 0.270 |
| AUROC | 0.669 (0.616–0.716) | 0.674 (0.621–0.722) | 0.684 (0.634–0.730) |
| AUPRC | 0.217 (0.165–0.286) | 0.221 (0.169–0.291) | 0.227 (0.180–0.296) |
| Brier score | 0.198 (0.188–0.207) | 0.209 (0.200–0.219) | 0.209 (0.200–0.219) |
| Sensitivity | 0.956 (0.900–0.985) | 1.000 (0.968–1.000) | 0.965 (0.912–0.990) |
| Specificity | 0.207 (0.178–0.238) | 0.005 (0.001–0.014) | 0.171 (0.144–0.200) |
| NPV | 0.969 (0.929–0.990) | 1.000 (0.398–1.000) | 0.969 (0.922–0.991) |
| Above-cutoff proportion | 0.814 (0.787–0.840) | 0.995 (0.988–0.999) | 0.847 (0.821–0.871) |

B. Completed-record sensitivity analyses

| **Metric** | **Frozen all-history** | **Remove source-window status** | **Follow-up exclusion** |
| --- | --- | --- | --- |
| Test patients | 991 | 991 | 966 |
| Test infections | 221 | 221 | 221 |
| Score cutoff | 0.310 | 0.320 | 0.310 |
| AUROC | 0.724 (0.685–0.765) | 0.727 (0.687–0.767) | 0.720 (0.681–0.761) |
| AUPRC | 0.532 (0.467–0.603) | 0.535 (0.469–0.604) | 0.539 (0.476–0.606) |
| Brier score | 0.185 (0.178–0.192) | 0.186 (0.180–0.193) | 0.183 (0.176–0.191) |
| Sensitivity | 0.914 (0.869–0.947) | 0.910 (0.864–0.944) | 0.882 (0.832–0.922) |
| Specificity | 0.227 (0.198–0.259) | 0.243 (0.213–0.275) | 0.260 (0.229–0.293) |
| NPV | 0.902 (0.851–0.940) | 0.903 (0.855–0.940) | 0.882 (0.832–0.921) |
| Above-cutoff proportion | 0.804 (0.778–0.829) | 0.791 (0.764–0.816) | 0.772 (0.744–0.798) |

Count-based proportions use exact binomial 95% intervals; discrimination and probability error use conditional percentile bootstrap intervals. Cutoff-dependent values refer to raw scores. AUROC, area under the receiver operating characteristic curve; AUPRC, area under the precision-recall curve; PPV, positive predictive value; NPV, negative predictive value.

Follow-up-exclusion analyses retained original development/test identities and refit the corresponding development models.

Supplementary Figure S1. Feature-stage discrimination in 991 held-out records (221 infections). a,b Pre-infusion XGBoost. c,d Post-infusion XGBoost. e,f Combined XGBoost. Shading indicates pointwise 95% bootstrap intervals (1,000 patient resamples); area intervals use the same resamples. Dashed lines represent chance ROC discrimination and prevalence for PR.

Supplementary Figure S2. Combined-model SHAP dependence for variables ranked 1-12 by mean absolute importance. Points show individual contributions from 1,000 training records; red diamonds indicate category means and curves indicate LOWESS trends. Pale gray bars show record counts on the right-hand n axis. NA denotes missing categories. Displayed category positions follow registry coding.

Supplementary Figure S3. Combined-model SHAP dependence for variables ranked 13-24 by mean absolute importance. Points show individual contributions from 1,000 training records; red diamonds indicate category means and curves indicate LOWESS trends. Pale gray bars show record counts on the right-hand n axis. NA denotes missing categories. Displayed category positions follow registry coding.

Supplementary Figure S4. Cross-disease calibration. a Raw common-feature scores. b Recalibrated probabilities estimated using the adult source cohort and applied unchanged to the pediatric/AYA B-ALL cohort (n=465; 102 infections). Mean raw model scores or recalibrated probabilities and observed infection proportions are shown in eight quantile groups with 95% exact binomial intervals, with fewer groups if tied scores prevent distinct groups. Dashed lines indicate ideal calibration. Target-cohort outcomes do not enter recalibration fitting.

Supplementary Figure S5. Descriptive decision-curve calculations. a,b All-history weighted ensemble. c,d Corrected Day+14 dynamic model. e,f Cross-disease common-feature model. Left panels show the full mathematical threshold range 0.01-0.99; right panels show an explicitly labeled 0.01-0.30 display zoom. Raw and development-recalibrated scores are compared with treat-all and treat-none references. Net benefit is TP/n minus FP/n multiplied by threshold/(1-threshold). Curves are conditional on the fitted scores and are descriptive; the plotted threshold range does not establish an intervention threshold or clinical benefit.
